# Validation of a computerized decision support system to review pharmacotherapy treatment: scheduling guidelines

**DOI:** 10.1186/s12911-022-01914-6

**Published:** 2022-06-30

**Authors:** Clávison Martinelli Zapelini, Dayani Galato, Graziela Modolon Alano, Karina Saviatto de Carvalho Martins, Silvana Cristina Trauthman, Alessandra Soares, Fabiana Schuelter-Trevisol, Daisson José Trevisol

**Affiliations:** 1 Postgraduate Program in Health Science, University of Southern Santa Catarina (UNISUL), Avenida José Acácio Moreira, 787, Tubarão, Santa Catarina 88704-900 Brasil; 2 Brasília University (UNB), Brasília, Distrito Federal Brasil; 3 Pharmacy School, University of Southern Santa Catarina (UNISUL), Tubarão, Santa Catarina Brasil; 4 Centro de Pesquisas, Clínicas Do Hospital Nossa Senhora da Conceição, Tubarão, Santa Catarina Brasil

**Keywords:** Pharmaceutical care, Clinical Pharmacy Information Systems, Pharmaceutical Services, Drug Utilization Review

## Abstract

**Background:**

The review of pharmacotherapy can be conceptualized as a service in which the drugs used by the patient are reviewed to control the risks as well as to improve the results of the drug therapy, detecting, solving, and preventing issues associated with the drug, readjusting the doses and times (schedule) so that the treatment is not incompatible or in duplicity.

**Methods:**

The aim of the study was to validate an intelligent information system, which was developed to assist the scheduling activity in the pharmacotherapy review. The system used the concept of Genetic Algorithms. To validate the system, hypothetical cases were elaborated considering various aspects of pharmacotherapy such as underdose, overdose, drug interactions and contraindications. These cases were tested in the system and were also analyzed by pharmaceutical experts with clinical and research experience in the pharmacotherapy review process. The degree of agreement between the assessments of the appointments carried out by the pharmaceutical specialists and by the system were measured using the Kappa index with a 95% confidence interval.

**Results:**

In detecting errors and make propositions, the system was able to identify 80% of errors, with pharmaceutical experts identifying between 20 and 70% of errors. In relation the results of kappa between the cases, the system had 87,3% of concordance, whereas the best pharmaceutical expert had 75,5% of concordance, considering the correct answer.

**Conclusion:**

It can be concluded that with the methodology used, the investigation met the objectives and confirmed the system is effective for pharmaceutical review process. There are indications that the system can help in the Pharmacotherapy review process, being able to find prescription errors as well as to establish times for the use of medications according to the patient’s routine.

## Introduction

According to the World Health Organization (WHO), more than half of all medicines marketed in the world are incorrectly prescribed or dispensed; as a result, more than 50% of patients don’t use them or use them incorrectly [1]. In Brazil, several factors contribute to this reality, including polypharmacy, which still does not have a standardized term in the scientific literature regarding the number of allowed drugs, the indiscriminate use of antibiotics, self-medication, prescription without clinical guidelines and dispensing not in compliance with the RUM (Rational Use of Medicines) [2].

One of the actions to reduce drug-related problems is the practice of pharmacotherapy review, in which the pharmacist evaluates prescriptions to find potential inconsistencies and suggest a strategy for the medication to be used by the patient, whether they are RX drugs that gave rise to the service or medications that are already in use, readjusting the doses and times (schedule) so that the treatment is not incompatible or in duplicity [2]. Therapeutic duplicity or incompatibility may increase the risk of adverse reactions and interactions. When detected the pharmacist or physician must adjust the therapeutic regimens, scheduling the drugs in use or change the prescription [3].

The review of pharmacotherapy can be conceptualized as a service in which the drugs used by the patient are reviewed to control the risks as well as to improve the results of the drug therapy, detecting, solving, and preventing issues associated with the drug. This assessment must be structured and carried out with the patient, aiming at increasing adherence and minimizing potential errors [4]. Several studies have shown that the review of pharmacotherapy is beneficial to patients and fosters the rational use of medication [4, 5]. However, the review of pharmacotherapy is quite difficult because the search for reliable technical information about the drugs is required. In addition, the process is complex due to its connection with the patients’ profile and the pharmacotherapy adopted. Thus, time is needed to perform such review, and sometimes the lack of quality information hampers this service performance [6].

The use of information systems facilitates a rapid search for information; however, for the system to design a strategy for the drug use, it is not sufficient to have the information available; one must list all the variables and use intelligence mechanisms to reproduce the reasoning of the pharmaceutical expert. Therefore, technology and algorithm selection are factors that deserve to be highlighted. Among the several options is the area of Artificial Intelligence (AI), which is composed of algorithms that simulate human intelligence, making inferences and learning from new problems. One of the AI techniques are the Genetic Algorithms (GA), which are used in optimization problems, that is, problems that search for the best solution among all possible ones [7].

Many authors using Decision Tree learning for helping clinical diagnosis of diseases or health complication. This is a system based on data models, it depends on involvement of users and the objective is resolves the problems unstructured or poorly structured [8, 9]. Intelligent systems, because they seek to get closer to human brain behavior to make the necessary inferences, can learn from specialists based on the representation of clinical reasoning through rules inserted in a database. Once trained, these systems are free of contextual factors that can induce errors, factors that are inherent only in human beings.

In this study, Bayesian learning algorithms and Decision Tree learning were evaluated, and the GAs are a good option to optimize the medication schedule for patients, because they are used for classical optimization problems and are widespread for optimizing schedules, in terms of computational technology. They can list all variables interfering with the process [7]. For other features of the system, there is no need to use intelligent algorithms, because binary solutions meet the needs. The binary solution is a type of computational language, which allows calculation and arithmetic operations, but cannot learn from situations.

The development of health information systems due to their interdisciplinary nature and the need for aggregation of different professional knowledge, requires a strict validation process so that all resources offered by the system be efficient and reliable [10–12]. The validation of computer systems involves usability, navigability, performance, and interface. In this study, we decided to carry out the validations related to the health area, that is, with a focus on content validation, construct validation and validation related to a criterion. The reliability of data collection instruments is also assessed [13–15]. Thus, to assist in the pharmacotherapy review process, this study aimed to validate an intelligent information system, which, in addition to making information available to pharmacists, could also propose the adjustment of schedules and doses, thus simulating the pharmaceutical expert’s reasoning through the use of genetic algorithms by developing a Decision Support System (DSS) to help review pharmacotherapy more quickly and respect scheduling rules in addition to considering drug information. The hypothesis was that the system was capable of reproducing the reasoning of the pharmaceutical experts and setting the schedules with the same quality standard.

## Methods

After the development of DSS, the validation process was divided into three methodological steps, guided by theoretical assumptions that refer to the validation and reliability of data collection instruments.

### Previous stage: system development

The system was developed based on AI techniques so that it could make inferences related to the variables and rules of Table 1, and thus, indicate possible prescription errors, as well as proposed schedules. The technique is based on an AI type called Evolutionary Computing in which GAs are the main concept. The system was developed using JAVA language with a Postgress database and WEB application resources, using our own servers.

**Table 1:** Rules defined for the development of the decision support system to review pharmacotherapy treatment

Rule	Description
No use (NU)	They represent situations of contraindication, that is, situations that may pose risk to the patient and should be avoided. For example: drug contraindicated for a special condition (pregnancy) or drugs with drug interactions being used concomitantly
Scheduling (S)	Scheduling rules represent the minimum time span between two or more medications, as well as the schedule of certain medications that should be set due to the patient's routine
Observations (O)	Instructions on how to administer the drug to maximize its effect. For example, standing for at least 20 min after taking a medicine

The drug information database was created by proponents of this study, based on the scientific literature [16, 17] with information being defined for its construction, such as drug name, ATC code, dose, indication, contraindications, significant interactions with food and other drugs. In addition, when using the DSS, patient information such as age, gender, physiological status (eg, pregnancy, liver and kidney problems) and other medications already in use must be provided.

**Fig. 1:**
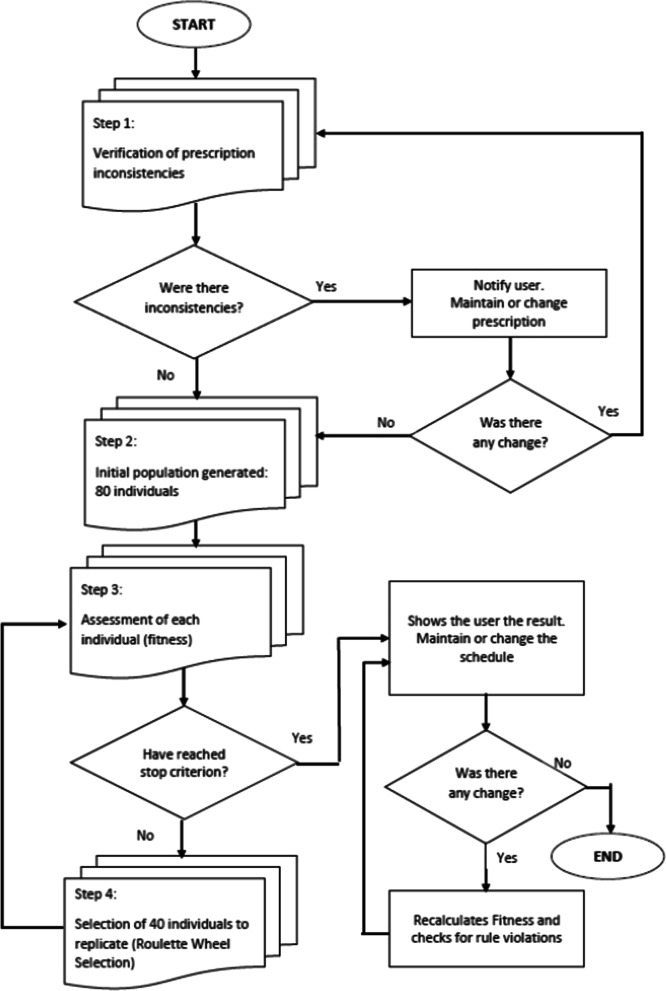
Decision Support System working process to review pharmacotherapy treatment

In this context, in addition to applying the rules described in Table 1, the system may create alerts to be examined by health professionals, in this case the system user. These alerts may be ignored after pharmaceutical experts’ analysis or considered for patient intervention. Alerts can indicate, for example, contraindications, interactions, therapeutic duplications, resulting from crossing information between the drug database and the patient's profile. If there are no alerts or when they are ignored by the user, the DSS presents the scheduling proposal that must be analyzed by the user who them modify the system if necessary. Because the system is developed through artificial intelligence, it can learn through interventions made by the users. Figure 1 summarizes how the DSS works. In other words, after the modification made by the users, the system considers the modification made as a proposed response that had not been proposed before. For example, when a user proposes that a particular drug should be used alone, the system begins to understand this information and, from there, new proposals will take this care into account.

### Validation process

In the first stage, the necessary records for the system’s operation were carried out; the system test was performed, and ten hypothetical cases were created that were sequentially coded (1–10) simulating patients with different routines and different drugs to be used. Both the patient's name and his/her routine were not real; however, they were based on situations that simulated daily life and covered several situations that involved different knowledge and skills for an adequate review of pharmacotherapy and scheduling. The records and cases were prepared by three professional pharmacists together with a professional who worked as a nurse, and the tests were performed by a professional in the computer field. Each case had some inconsistency or specific analysis situation, according to Table 2. In this case, it was the first verification made by the system. These inconsistencies were kept confidential at all stages of validation. In the first stage, the necessary registrations were carried out for the functioning of the system. They were made manually by three pharmaceutical professionals together with a nurse, specifically to examine 10 hypothetical cases that were created. The hypothetical cases were sequentially coded (1–10) simulating patients with different routines and different medications they should take. Neither the patient's name nor his/her routine was real; however, they were based on situations that simulated daily life and contemplated several situations that involve different knowledge and skills for the adequate review of pharmacotherapy and scheduling. As a basis for the analysis, we examined the medical records of the consultations carried out in 2015 at the Pharmaceutical Care Center of the University of Southern Santa Catarina. The hypothetical cases purposely had situations of inconsistency or situations that require specific analysis in the process, according to Table 2. These inconsistencies were kept confidential at all stages of the validation process.

**Table 2:** Predicted situations for hypothetical cases

Analysis situation	Hypothetical cases
Medicine that should not be used concurrently	“ACETYL-SALICYLIC ACID” and “WARFARIN” present in drugs “ASPIRIN 500 mg” and “WARFARIN 2.5 mg” in the same prescription
Prescription for patients who have an active daytime routine	Patient who wakes up at 7am, work from 8am to 12 pm and from 1 to 6 pm. Sleeps at 10 pm
Prescription for patients who have an active night routine	Patient who works from 10 pm to 6am and sleeps from 7:30am to 2:30 pm
Medicines that require a minimum interval between doses	Use of “DIGOXIN” and “MYLANTA PLUS®” which have interaction between active principles “DIGOXIN” and “ALUMINUM HYDROXIDE” requiring an interval between doses
Prescription for a patient who has a health condition in which a drug is contraindicated	Patient with severe liver disease (cirrhosis) with prescription of “ATORVASTATIN CALCIUM” which is contraindicated drug for this situation
Overdose prescription for a particular drug	2-years-old child weighing 13 kg with a prescription of the “AZITROMYCIN” with a dose higher than the maximum dosage allowed for the child’s weight
Prescription of medications that need interaction with food	Prescription of “OMEPRAZOLE” which is indicated to be used on an empty stomach
Prescription for patients in which sleep disruption should be avoided	6-years-old child who needs antibiotics every 8 h
Medicine with indication not to be used, but by clinical decision opted for the risk	“CARBAMAZEPINE” for a pregnant woman with 12 weeks of gestation, to avoid epilepsy crises
Medicines that need specific guidelines for use	Use of “ALENDRONATE SODIUM” and “LEVOTHYROXINE”

In the second stage, the simulated cases were submitted to the pharmacotherapy panel for review and the schedule was established by five specialized pharmacists. In parallel, those cases were also scheduled by the system, generating a total of six schedules for each case. The pharmaceutical experts took 109 min in average to schedule the 10 cases submitted.

**Fig. 2:**
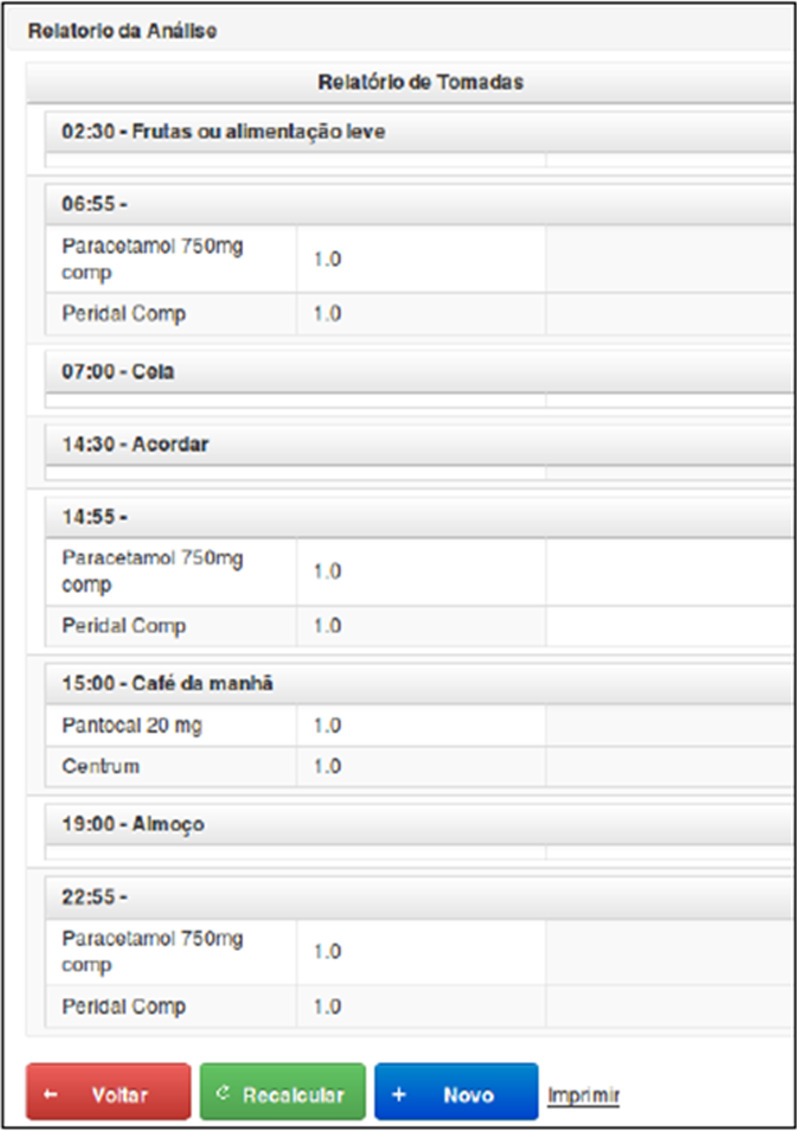

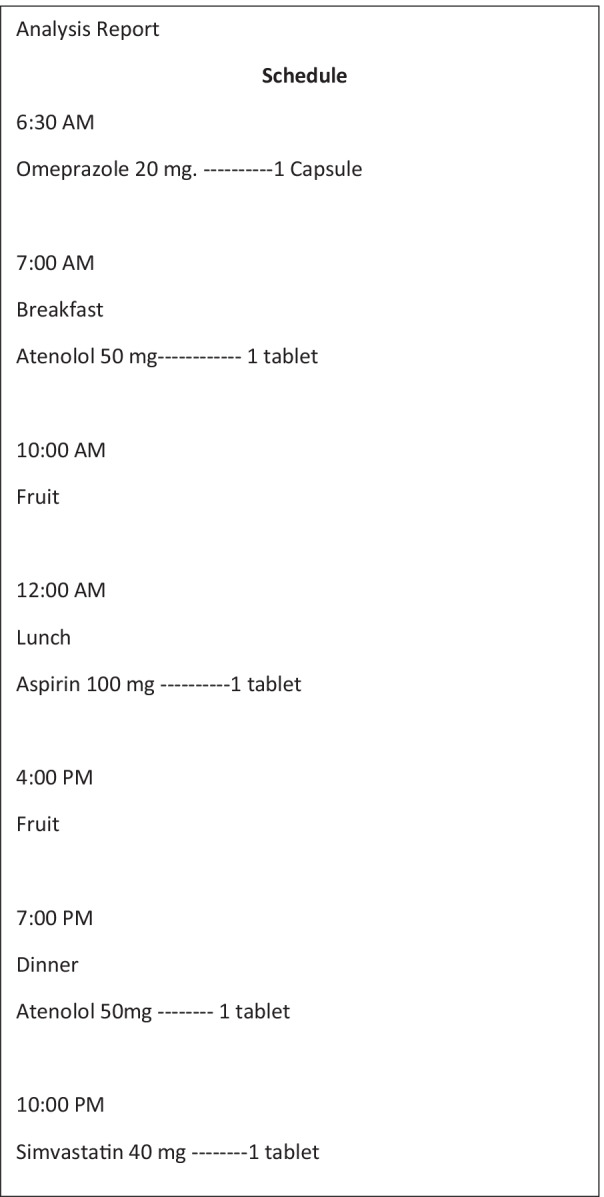
Register of pharmacological interactions (original and translated)

Pharmacists filled out a standardized table that contained the schedule they recommended, the name of the drug, the dosage, as well as a space for them to enter notes next to each drug, if they thought necessary, such as instructions on the use, prescription reassessment recommendation, among others (Table 2) and Fig. 2.

In the pharmacotherapy review performed by pharmacists, the purposefully created inconsistencies were expected to be discovered and highlighted in a specific space on the form. Appointments made by pharmacists were identified by a number (1–5) and were called human experts (terminology used in the computational area to differentiate the human expert from the system expert), in this case pharmaceutical experts. The cases were also submitted for scheduling by the developed system, which, in addition to adjusting the schedules to better suit the patient's routine, had the functionality to issue alerts about overdose inconsistencies, drugs that should not be used concomitantly, drugs requiring a minimum interval between doses, prescription alerts for patients who have a health condition in which a particular drug is contraindicated, such as pregnancy, alerts for the prescription of drugs that need interaction with food, and observations on drugs that need specific guidelines to be used. The system was handled by an expert who was not involved in any other validation step and the system results were transcribed into a table the same way as the one filled in by the pharmaceutical experts. This step was important so that future validators could not distinguish between scheduling performed by the system and by pharmaceutical experts, thus avoiding possible biases. For the other validation steps, the system was treated as an additional expert, assigned as number six. The process of entering data into the system and issuing the report took 10 min, since all the data were already registered in step 1.

In addition to the information in the table, which was filled in for each drug, the form contained a space for justification regarding the schedule made, or not made for some reason. In this justification, the pharmacist could include information that he/she would deem relevant for the scheduling, such as prescription errors that should be revised, observations of inconsistency or suggestions for the adequacy of medications. For the last validation step, pharmacists were identified by a number (1–5) and were called human experts.

The cases were also submitted for scheduling to be carried out by the system; such system was handled by a non-expert human professional who was not involved in any other validation stages. The latter used the system developing the schedules and transcribed the results presented by the system in a table the same way as the one filled in by the human experts. This step was important so that future validators would not be able to distinguish the schedules set up by the system from the schedules set up by the human experts, thus avoiding potential biases. For the other validation steps, the system was treated as an additional expert, receiving the number six.

In the third and final stage, all reviews and schedules carried out, both by human and system experts, were evaluated and validated through consensus by five professionals with extensive clinical and research experience in the pharmacotherapy review process. These specialists are referred to in this study as “Validator Experts” (VEs). A blinding process was used, so that the VEs did not know which schedule had been set by a pharmacist or by the system, as they had not participated in the previous stages of the system development and registration. The classification of the VEs, which established whether the review and scheduling are considered adequate (all scheduling is correct), partially adequate (part of drug scheduling is adequate, and the situation don’t compromise the clinical results), or inadequate (all are incorrect or the propose probably compromising the clinical results).

For the data collection of the schedules’ quality indicators a data collection instrument translated from the *Índice de Adequação de Medicação* (IAM, Medication Adequacy Index) was used. This instrument was developed by Hanlon et al. [19] based on a literature review on assessment measures or medication assessment scales. Subsequently, the IAM was validated by Samsa et al. [20]. After the translation, the instrument was called *Índice de Avaliação da Revisão da Farmacoterapia* (IARF, Pharmacotherapy Review Assessment Index) and is presented in Table 3.

**Table 3:** Pharmacotherapy review assessment index (IARF). *Source* Adapted from Hanlon et al. [19]

1	Are there any medications that are not indicated for the patient?	1 No	2 Yes, but acceptable	3 Yes	9 I do not know
2	Are there any medication that could have been replaced by a more effective one?	1 No	2 Yes, but acceptable	3 Yes	9 I do not know
3	Is any medication in the wrong dosage for the patient?	1 No	2 Yes, but acceptable	3 Yes	9 I do not know
4	Are any medication specified with incorrected or inadequate administration?	1 No	2 Yes, but acceptable	3 Yes	9 I do not know
5	According with patient’s routine, is there any medication in which the hours of use are inadequate?	1 No	2 Yes, but acceptable	3 Yes	9 I do not know
6	Does any medication have drug interaction that can cause harm for the patient?	1 No	2 Yes, but acceptable	3 Yes	9 I do not know
7	Are there any medication that should do not be used due the special conditions or clinical situations of the patient?	1 No	2 Yes, but acceptable	3 Yes	9 I do not know
8	Is there unnecessary duplication of prescription?	1 No	2 Yes, but acceptable	3 Yes	9 I do not know
9	Are there any medications in which the minimum time between doses is a risk for the patient?	1 No	2 Yes, but acceptable	3 Yes	9 I do not know
10	Overall, you consider this review of pharmacotherapy	1 Adequate	2 Partially adequate	3 Inadequate	9 I do not know

The questions were prepared in such a way that the lowest score answer (1—No) will always be optimal and the sum of the answers, for an optimal situation, should attain a maximum of 10 points.

The degree of agreement between the schedules assessments that were carried out by the pharmaceutical experts and by the system was measured using the Kappa index with a 95% confidence interval.

This study was performed in accordance with the Declaration of Helsinki and approved by the Research Ethics Committee of the University of Southern Santa Catarina (UNISUL) under CAAE number 20992713.60000.5369 and under Opinion number 461.125.

## Results and discussion

With the IARF of each hypothetical case, the aspects that cause the most errors in the Pharmacotherapy Review process were verified. Three aspects were selected to be analyzed: the identification of intentional errors, the adequate adjustment of medication usage schedule and the final classification of the VEs, which established whether the review and scheduling are considered adequate, partially adequate, or inadequate.

**Fig. 3:**
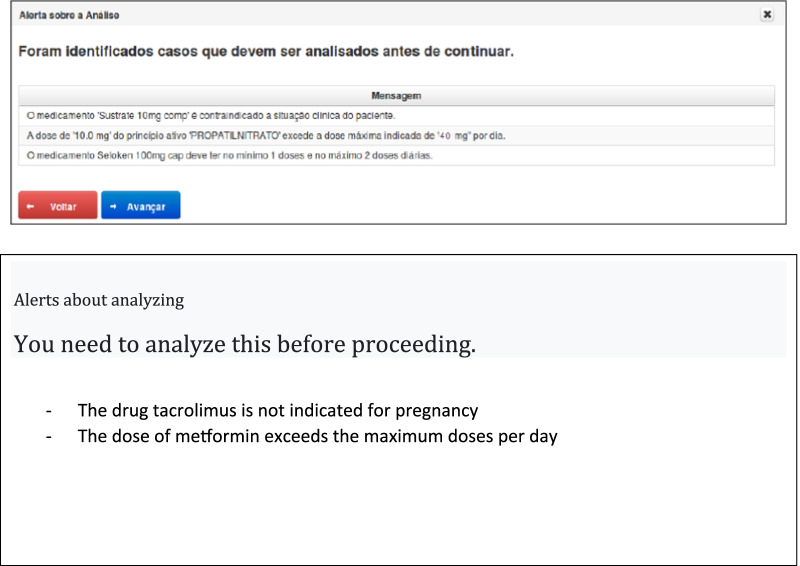
Alerts screen

In these three aspects of evaluation, the system obtained a greater number of correct answers as compared to those of the pharmaceutical experts. The intentional errors by the system was not identified in the hypothetical case where a drug that is not indicated for the patient's clinical condition appears in the prescription. The reason is because the system does not automatically remove the drug from the prescription; it only warns and leaves the decision to the user. The other case was the prescription of the drug “alendronate sodium”: the VEs considered that the directions on the use of this drug should be more detailed when related to the schedule and include information based on the Ministry of Health clinical protocol [21]. For these two situations, adjustments in the registration of medications would resolve the negative assessment of the review by the system. [Fig. 3].

The system issued alerts for all intentional errors that were present in the hypothetical cases, but it did not automatically withdraw the drug from the prescription. In one hypothetical case, there was a prescription for the drug “alendronate sodium”, the EVs considered that the observations on the use of this drug should be more detailed when related to the scheduling and have information based on the clinical protocol of the Ministry of Health [21]. For that situation, adjustments in the medication register would resolve the observation generated by the system.

Regarding the schedule adjustments, those set up by the system were the only ones that were considered adequate in 100% of the reviews. Therefore, the selection of GA as the main system intelligence resource proved to be efficient in adjusting the medication use schedule. Optimization problems, in general, have a finite space of solutions and restrictions for solutions to be considered about adequate. To carry out the scheduling with the selection of the most adequate times for the medicine use, the finite space for solutions is constituted by the hours of the day and the medicines that need to be scheduled. The restrictions are the patient's routine impositions and the interactions between medications and between medications and food that sometimes end up causing the patient to abandon treatment [20]. In some cases, there are a lot of restrictions related with patient routine, for example the work time or another compromise, these situations are not problem for the algorithms to finding a solution. The GAs, even without finding an optimal solution, show the best solution found given the restrictions related to the patient's routines.

Finally, in the last classification of the pharmacotherapy review, the reviews made by the system were those that obtained the best ratings, being considered adequate (88%). The pharmaceutical expert who came closest to this rate obtained 75% of the reviews considered adequate (Table 4).

**Table 4:** IARF assessment results

Revisor	Identification of intentional erros (%)	Proper schedule adjustment (%)	Final classification partially		
			Adequate (%)	Adequate (%)	Inadequate (%)
Specialist 1	20.0	40.0	65.0	10.0	25.0
Specialist 2	40.0	80.0	73.0	7.0	20.0
Specialist 3	30.0	60.0	62.0	10.0	28.0
Specialist 4	50.0	70.0	69.0	9.0	22.0
Specialist 5	70.0	80.0	75.0	11.0	14.0
Electronic system	80.0	100.0	88.0	6.0	6.0

To establish agreement between the reviews carried out by the pharmaceutical experts and the reviews carried out by the system, the indices presented in Table 5 were obtained. The reviews carried out by the pharmaceutical experts were called A1, A2, A3, A4 and A5 and the review performed by the system was called A6.

**Table 5:** Agreement rates between pharmaceutics experts and electronic system among pharmacotherapy review

	Weighted Kappa	CI 95%
A6–A1	0.3547	(0.1926–0.5168)
A6–A2	0.3719	(0.1823–0.5615)
A6–A3	0.3458	(0.1907–0.501)
A6–A4	0.3971	(0.2241–0.5701)
A6–A5	0.4790	(0.2759–0.6822)

*CI *confidence level

The agreement rate obtained was considered reasonable (0.21–0.40). The highest agreement occurred with the A5 schedule and was considered moderate (0.41–0.60). The results obtained indicate that the system exceeded the quality of the scheduling performed by pharmaceutical experts, mainly in the identification of prescription errors and adequacy of schedules according to the patients' routine, in addition to the fact that once the data was registered, the task execution time had a significant reduction. The low levels of agreement were then interpreted as positive, since the reviews performed by the system were better evaluated qualitatively than reviews performed by pharmaceutical experts.

The initial hypothesis was that the system was capable of reproducing the reasoning of the pharmaceutical experts and setting the schedules with the same quality standard. However, the results obtained showed that the system surpassed the schedule quality performed by pharmaceutical experts, mainly in the identification of prescription errors and adequacy of schedules in connection with the patients' routine. The low levels of agreement were then interpreted as positive, since the revisions carried out by the system were better evaluated qualitatively.

The number of pharmaceutical experts who set the schedules to be compared with the schedules set by the system (5) and the number of simulated hypothetical cases (10) are not sufficient to establish a statistically significant sample, which was a limitation of this study. However, it was possible to verify that the system was able to set schedules with a standard of equal quality and even superior to those of the experts who participated in this study. This according to the evaluation carried out by the VEs.

Establishing the system's reliability index would be a feature that could be used to validate the system for reproducibility, that is, to validate multiple schedules of the same hypothetical case, performed by the system, at different times, and verify that all these schedules would be classified as "adequate". This analysis was not performed because the only way to assess whether the schedule is adequate or not, in the methodological parameters that were used, would be through the validation performed by the VEs in the consensus meetings, which would demand an excessive time for analysis thus making the process unfeasible.

It is noteworthy that the system only obtains good results if the registrations are carefully performed. The intelligent reasoning provided by GA will only work if the database has the correct relationships, including writing patterns and quality of observations. The cases in which the system did not obtain the expected result included situations in which the adjustment in the medication record would have cured the fault indicated by the EVs. In the pharmaceutical care settings where medications are dispensed, the main difficulties found for the pharmacotherapy review service to be fully carried out are the lack of quality information and the excessive time required to look for prescription potential inconsistencies and for suggesting a drug use strategy considering the patient's routine.

Failure to carry out the review of pharmacotherapy as recommended by the WHO has a direct impact on the RUM promotion, since the pharmacist, through a set of actions called pharmaceutical care (PC), which includes pharmacotherapy review, is an extremely important agent in promoting the RUM, interacting between the prescriber and the drug end user.

The fast search for information can be performed using an information system; however, in addition to having the information available, it is necessary to handle it, to establish the adjustment of doses and schedule consistent with the patient's routine; hence, this system can be used as a resource to support decisions, with the final decision always being the responsibility and technical competence of human beings.

## Conclusion

It can be concluded that with the methodology used, the investigation met the objectives and confirmed the initial hypothesis, since the system was able to produce results considered adequate by the VEs, being able to set quality schedules, as fast as the computerization processes allow, in addition to the security of producing results without interference of contextual factors to which human beings are susceptible.

The fact that the system was able to identify the highest rate of intentional errors (80%), surpassing all pharmaceutical experts, proves that once the database (inference base) is correctly modeled and registered, it will never cease to identify the errors, and it is up to the pharmaceutical experts to make the clinical decisions, based on further information that is not part of the scope of the system.

## Data Availability

The data that support the findings of this study are available, but restrictions apply to the availability of these data, which were used under license for the current study, and so are not publicly available. Data are however available from the authors upon reasonable request and with permission of: https://riuni.unisul.br/bitstream/handle/12345/4838/Tese%20RIUNI.pdf?sequence=4&isAllowed=y
